# Application of layered double hydroxides as laccase mimicking nanozymes in the oxidation of 2,3-dihydroquinazolin-4(1*H*)-ones and hantzsch 1,4-dihydropyridines

**DOI:** 10.1039/d5ra03505h

**Published:** 2025-09-30

**Authors:** Nadia Ghorashi, Amin Rostami

**Affiliations:** a Department of Chemistry, Faculty of Science, University of Kurdistan Sanandaj 66177-15175 Iran a.rostami@Uok.ac.ir +988716624004 +989183730910

## Abstract

This study explores the innovative use of copper–manganese–iron layered double hydroxides nanozyme (CuMnFe-LDHzyme) in aerobic oxidation of *N*-heterocyclic compounds. The successful fabrication of CuMnFe-LDHzyme was substantiated through X-ray powder diffraction (XRD) analysis. Energy-dispersive X-ray (EDX) spectroscopy was employed to ascertain the presence of the metal ion composition. Morphological alterations were investigated using scanning electron microscopy (SEM). Inspired by the laccase-mediated system, herein the combination of CuMnFe-LDHzyme with 2,3-dichloro-5,6-dicyano-1,4-benzoquinone (DDQ) or 4-phenyl urazole (4-PU) as a cooperative catalytic system was used for the oxidative synthesis of quinazolinones (60–95% yield) and pyridines (65–95% yield) using O_2_ as ideal oxidants in acetonitrile at 50–60 °C. The CuMnFe-LDHzyme not only mimics the functionality of laccase enzymes but also enhances catalytic activity through its unique layered structure, facilitating electron transfer and increasing reaction efficiencyWe believe that synthesized CuMnFe-LDHzyme superior tolerance to variations in temperature, ionic strength, and storage conditions, as well as excellent recyclability compared to naturally occurring laccase counterparts.

## Abstract 2

The aerobic oxidation of 2,3-dihydroquinazolin-4(1*H*)-ones and hantzsch 1,4-dihydropyridines in the presence of layered double hydroxides as laccase mimicking nanozymes is describe.

## Graphical abstract

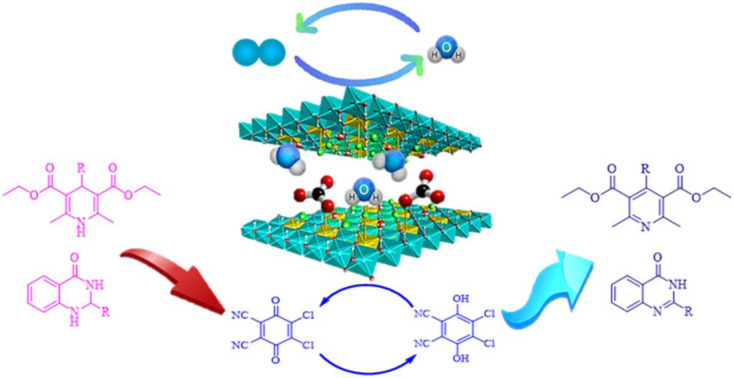

## 1. Introduction

Natural enzymes are essential for facilitating biochemical reactions, demonstrating remarkable activity and selectivity. Laccase, in particular, is one of the most extensively studied and versatile enzymes, capable of oxidizing various substrates, including phenolic compounds such as polyphenols, aminophenols, and polyamines.^1,2^ The catalytic mechanism of laccase involves the oxidation of substrates through the reduction of molecular oxygen to water, aided by a multi-copper active site. However, traditional laccase often suffers from poor stability in complex environments. Its inherent limitations, including low stability, high purification costs, sensitivity of catalytic activity to environmental conditions, and challenges in recycling and reuse, significantly hinder its widespread application in *in vitro* settings and practical industrial contexts.^3^ As a results, enzyme mimics, also known as “artificial enzymes”, have gained attention as potential substitutes for natural enzymes.^4^ Among these, nanozymes have arisen as the next generation of enzyme mimics. Nanozymes, defined as nanomaterials with inherent enzymatic properties, constitute an innovative class of artificial enzymes that have sparked significant interest owing to their exceptional attributes. Nanozymes exhibit various distinct features that render them highly appealing for different applications, such as high stability, high activity, easy production procedures, low cost, reusability, and versatility,^5^ potentially yielding profound implications in various scientific and industrial realms. Due to their remarkable characteristics, nanozymes have surfaced as compelling substitutes for natural enzymes in areas including biomedical applications, environmental remediation,^6^ industrial processes, and more. Various nanomaterials have been discovered to exhibit enzyme-like behaviors, including noble metals,^7^ metal oxides,^8^ carbon nanomaterials,^9,10^ metal–organic frameworks (MOFs),^11^ and layered double hydroxides (LDHs).^12^ Layered double hydroxides (LDHs), also known as anionic clays or hydrotalcite-like clays, are a category of materials characterized by a stratified arrangement.^13^ These LDHs represent a type of planar material constituted by the recurring arrangement of positively charged octahedral MO_6_ host layers accompanied by negatively charged anions and water molecules inserted among these layers.^14^ The distinctive attributes of LDHs, such as their considerable surface area, adjustable interlayer spacing, and capacity to accommodate diverse guest molecules or ions, have garnered considerable interest. LDHs have been found to have utility across various domains, including but not limited to catalysts,^15,16^ drug delivery systems,^17^ flame retardants,^18^ and biomaterials.^19,20^

Recently, several scholarly investigations have shown that Layered Double Hydroxides (LDHs) are promising candidates as enzyme mimics, owing to their substantial specific surface area, abundant redox reaction active sites, adjustable morphology, and dimensions, in addition to their biocompatibility. Thus far, several LDH-based nanoenzymes (LDHzymes) have been reported.^21,22^ The catalytic activity of natural laccase hinges on the presence of copper ions, which serve as a crucial component of its active site.

Consequently, it is hypothesized that incorporating copper into the artificial enzymes designed to laccase mimic functionality could lead to improved performance. Given the low redox potential, native laccases can only oxidize electron-rich aromatic substrates. Previous research has shown that one way to enhance laccase activity is through the use of mediators.^23^ Common artificial mediators used in studies of laccase-like catalysis include TEMPO (2,2,6,6-tetramethylpiperidine-1-oxyl),^24^ DDQ (2,3-dichloro-5,6-dicyano-1,4-benzoquinone),^25^ and ABTS(2,20-azino-bis(3-ethylbenzthiazoline-6-sulfonate).^26^ Inspired by laccase-mediated systems, the combination of LDHzyme with mediators offers a strategic approach for extending their applications in organic chemistry.

2,3-Dichloro-5,6-dicyano-1,4-benzoquinone (DDQ) is the most widely used quinone due to its high reduction potential.^27^ It facilitates hydride transfer reactions and exists in three accessible oxidation states: quinone (oxidized), semiquinone (one-electron-reduced), and hydroquinone (two-electron-reduced).^28^ DDQ is extensively employed as a stoichiometric oxidant in the dehydrogenation of saturated C–C, C–O, and C–N bonds.^29^ However, its high toxicity, cost, and the challenges related to isolating by-products, such as DDQH_2_, pose significant obstacles to its large-scale application. To address these issues, methods have been developed that utilize a catalytic amount of DDQ alongside a less expensive co-oxidant capable of regenerating DDQ from its reduced hydroquinone form.^30^ Recently, catalytic oxidation systems employing small amounts of DDQ as mediator and a co-catalyst, with the presence of molecular oxygen as a terminal oxidant, have attracted more attention.^25,31^

4-Phenyl-1,2,4-triazole-3,5-diones have been introduced as efficient oxidative reagents due to their dehydrogenating properties. However, the use of stoichiometric amounts of 4-phenyl-1,2,4-triazole-3,5-diones as effective oxidizing agents is limited by their low stability, high cost, and toxicity, which restricts their application in organic synthesis. The most effective approach to overcoming these challenges is to generate 4-phenyl-1,2,4-triazole-3,5-diones *in situ* from stable, inexpensive, and non-toxic urazoles. 4-Phenylurazole is stable and can be oxidized to produce 4-phenyl-1,3,4-triazole-3,5-dione.^32,33^

Quinazolin-4-(3*H*)-one is representative of a distinct class of annulated six-membered nitrogen heterocycles and serves as a fundamental structural component in a variety of natural products and biologically active substances. Due to their pervasive presence and critical role as pharmacophores in potential therapeutic agents, they are categorized as privileged structures.^34^ These compounds exhibit a plethora of pharmacological and biological properties, including but not limited to anticancer,^35^ antimalarial,^36^ antihypertensive,^37^ anti-inflammatory,^38^ and antitubercular activities.^39^ In light of their extraordinary importance, substantial efforts have been directed towards the development of efficient and pragmatic methodologies for the synthesis of the quinazoline framework, particularly focusing on 2-substituted quinazoline.

Pyridines also represent a noteworthy category of heterocyclic compounds. Their significance encompasses various applications, such as in natural products, flavourings, fragrances, pharmaceuticals, agrochemicals, dyes, and polymers. The intrinsic structural characteristics of pyridines contribute to their utility as ligands in coordination chemistry, where they promote interactions with metallic centers, and as essential reagents and foundational components in the vast domain of organic synthesis.^40,41^

In continuation of our systematic research about laccase-mediated catalytic system in aerobic oxidation of organic compounds,^42,43^ to overcome some limitation with laccase, in this work, for the first time, CuMnFe-LDHzyme in the combination with 2,3-dichloro-5,6-dicyano-1,4-benzoquinone (DDQ) or 4-phenyl urazole as a cooperative catalytic oxidation system were used for the aerobic oxidative synthesis of 2,3-dihydroquinazolin-4(1H)-ones and Hantzsch 1,4-dihydropyridines.

## 2. Result and discussion

### 2.1. Preparation and characterization of CuMnFe-LDHzyme

**Fig. 1:**
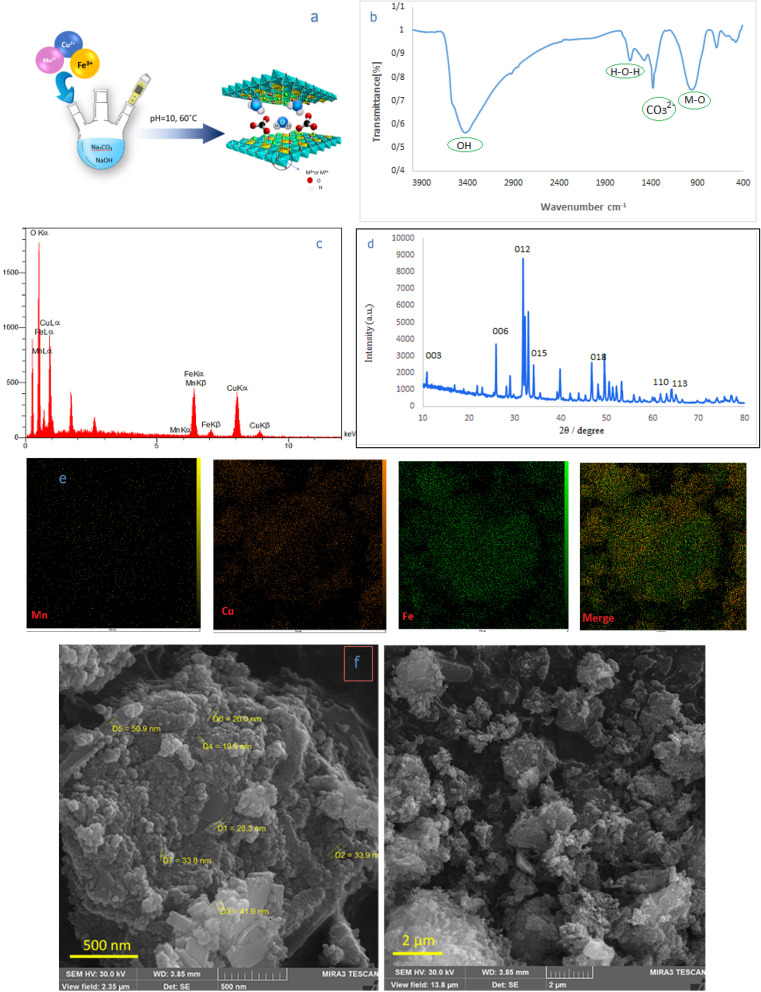
(a) Illustrates a typical fabrication procedure for CuMnFe-LDHzyme. (b) FT-IR spectra of CuMnFe-LDHzyme. (c) EDS analysis (d) XRD patterns of the CuMnFe-LDHzyme. (e) Mapping of the CuMnFe-LDHzyme catalyst. (f) SEM characterization of the CuMnFe-LDHzym catalyst.

The synthesis of CuMnFe-LDHzyme is accomplished through a straightforward co-precipitation method.^44^ The co-precipitation method involves the simultaneous precipitation of metal ions from a solution, leading to the formation of layered double hydroxides (Fig. 1a). This technique is advantageous due to its simplicity, cost-effectiveness, and ability to produce materials with high purity. During the synthesis, copper, manganese, and iron ions are mixed in predetermined ratios, followed by the addition of a precipitating agent, which induces the formation of the desired laccase-like enzyme structure. The CuMnFe-LDHzyme was characterized using FESEM, EDS, FT-IR, ICP and XRD. To evaluate the structural fidelity and phase purity of the produced LDHzyme, analysis of functional groups on the surface of Layered Double Hydroxides (LDHs) through Fourier Transform Infrared (FT-IR) spectroscopy provides critical insights into their structural and chemical properties (Fig. 1b). The FT-IR spectra reveal several significant features, particularly within the broad band observed between 3200 and 3650 cm^−^^1^ This band is attributed to the O–H stretching vibrations, which originate from both the metal hydroxide layer of the LDHs and interlayer water molecules. In addition to O–H stretching, the bending vibrations of the interlayer water molecules are evidenced by absorption peaks near 1637 cm^−1^. This further illustrates the involvement of water in the interlayer space, which is essential for the stability and functionality of LDH materials. Notably, the distinct peak at approximately 1356 cm^−1^ corresponds to the telescopic vibrations of the carbonate ion (CO_3_^2−^), indicating its successful incorporation into the interlayer structure of the LDHs. Furthermore, the spectral region encompassing 700 to 1100 cm^−1^ features peaks associated with lattice vibration modes, such as M–OH, M–O–H, and M–O bonds.^45^

These peaks are consistent with the fundamental characteristics of LDHs, reinforcing our understanding of their layered structure an interlayer interaction.

X-ray diffraction (XRD) analysis was conducted. The obtained XRD patterns revealed a characteristic set of 2 theta angles, which are representative 10.5°, 24.5°, 34.1°,39.7°,46.2°,61.6° and 63.2°indicating typical LDH characteristics (Fig. 1d).^44,46^ Scanning electron microscopy (SEM) provides valuable data on the morphology and particle distribution of the LDHzymes (Fig. 1f). SEM images in Fig. 1 shows that the lateral size of the obtained CuMnFe-LDHzyme was approximately 19–50 nm.

This measurement underscores the uniformity and consistency in particle size. Energy-dispersive X-ray (EDX) mapping analyses conducted on the CuMnFe-LDHzyme revealed that the elements Cu, Mn, and Fe exhibited a homogeneous distribution throughout the sample (Fig. 1c). Moreover, the ICP spectrum showed that the atomic percent elements of Cu, Fe and Mn were 34.63%, 15.33% and 7.3%. The long-term storage stability of the CuMnFe-LDHzyme was also investigated.

### 2.2. Investigation of the catalytic efficacy of the CuMnFe LDHzyme's

The laccase-like catalytic activity of CuMnFe-LDHzyme was surveyed to examine concerning the aerobic dehydrogenation of 2-substituted-2,3-dihydroquinazolin-4(1*H*)-ones and 1,4-dihydropyridines.

Initially, the catalytic application of LDHzymes (140 mg) for the aerobic oxidation of 2-phenyl 2,3-dihydroquinazolin-4(1*H*)-one in the presence of DDQ (10 mol%) as mediator in CH_3_CN at 60 °C was selected as a model reaction (Table 1). Different LDHzyme were tested (Table 1, entries 1–5). Among them, CuMnFe-LDHzyme exhibited the highest catalytic efficiency (Table 1, entry 2).

**Table 1:** Optimization catalysts and mediators for aerobic oxidation reaction conditions of 2-phenyl 2,3-dihydroquinazolin-4(1*H*)-one^a^

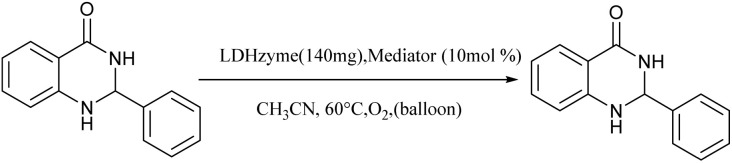			
Entry	LDHzyme	Mediator	Yield (%)
1	CuFeZn-LDHzyme	DDQ	65
**2**	**CuFeMn-LDHzyme**	**DDQ**	**98**
3	CuFe-LDHzyme	DDQ	50
4	MnFe-LDHzyme	DDQ	57
5	MnCu-LDHzyme	DDQ	68
6	—	DDQ	45
7	CuFeMn-LDHzyme	—	55
8	CuFeMn-LDHzyme	DDQ	70^b^
9	CuFeMn-LDHzyme	4-Tert	45
10	CuFeMn-LDHzyme	DTBC	55
**11**	**CuFeMn-LDHzyme**	**4-Phenyl urazole**	**98**

a Reaction conditions unless stated otherwise: substrate (1 mmol), LDHzyme (140 mg), mediator (10 mol%), CH_3_CN (3 mL) under O_2_ (balloon), and 18 h; the bolds represent the effective reaction conditions.

b The reaction was performed under open flask.

Furthermore, it is pertinent to highlight that the aerobic oxidation of 2-phenyl-2,3-dihydroquinazolin-4(1*H*)-one in the absence of either LDHzyme, DDQ or molecular oxygen (baloon) lead to low yield of desired product (Table 1, entries 6–8). These findings unequivocally indicate that the successful execution of this transformation necessitates the synergistic action of CuMnFe-LDHzyme, DDQ, and molecular oxygen (O_2_). Various other mediators were also investigated for on the model reaction. The DDQ and 4-phenyl urazole provided the most promising results (Table 1, entries 2 and 11), while catechol derivatives such as 4-*tert*-butylcatechol and DTBC led only to low or moderate yields.

We also tested the effect of different types of parameters, including the amount of CuFeMnLDHzyme and mediator (DDQ and 4-phenyl urazole), temperature, and solvent, on the aerobic oxidation of 2-phenyl-2,3 dihydroquinazolin-4(1*H*)-one as model compound. The findings unequivocally revealed that a reduction in the quantities of DDQ(5 mol%), 4-phenyl urazole (5 mol%), CuFeMnLDHzyme(110 and 130 mg) and temperature(45 °C) resulted in a corresponding decrease in product yield (Table 2, entries 1–10). Among the various solvents screened, CH_3_CN gave the best result (Table 2, entries 11–14).

**Table 2:** Optimization of reaction conditions for aerobic dehydrogenation of 2-phenyl-2,3 dihydroquinazolin-4(1*H*)-one using CuMnFe-LDHzyme/mediator catalytic system^a^

Entry	Mediator (mol%)	Amount of CuMnFe-LDHzyme	Temperature(°C)	Solvent	Yield (%)
1	DDQ (5)	140	60	CH_3_CN	72
**2**	4-Phenyl urazole(5)	140	60	CH_3_CN	70
3	**DDQ (10)**	**140**	**60**	**CH**_**3**_ **CN**	**98** b
**4**	**4-Phenyl urazole(10)**	**140**	**60**	**CH**_**3**_ **CN**	**98** b
5	DDQ (10)	110	60	CH_3_CN	68
**6**	4-Phenyl urazole (10)	110	60	CH_3_CN	65
7	DDQ (10)	130	60	CH_3_CN	85
**8**	4-Phenyl urazole(10)	130	60	CH_3_CN	80
9	DDQ (10)	140	45	CH_3_CN	70
10	4-Phenyl urazole(10)	140	45	CH_3_CN	75
11	DDQ (10)	140	60	CH_3_CN/H_2_O	35
12	4-Phenyl urazole(10)	140	60	CH_3_CN/H_2_O	30
13	DDQ (10)	140	60	DMSO	60
14	4-Phenyl urazole(10)	140	60	DMSO	60

a Reaction conditions unless stated otherwise: substrate (1 mmol), CuMnFe-LDHzyme (140 mg), mediator (10 mol%), solvent (3 mL) under O_2_ (balloon), and 18 h;

b The bolds represent the effective reaction conditions.

Under the optimized conditions (Table 2, entries 3 and 4), we investigated the dehydrogenation of various 2-substituted-2,3-dihydroquinazolin-4(1*H*)-ones. The findings are summarized in Table 3. The expected products were synthesised at commendable yields. Notably, the inclusion of electron-donating groups significantly enhanced the reaction rate, regardless of their positional arrangement (Table 3, entries 2–4). Additionally, 2-substituted-2,3-dihydroquinazolin-4(1*H*)-one derivatives incorporated electron-withdrawing groups yielded the corresponding 2-substituted quinazolin-4(3*H*)-ones in exemplary yields (Table 3, entries 5–9).

**Table 3:** Synthesis of quinazolinone derivatives *via* aerobic oxidation using CuMnFe LDHzyme/DDQ and CuMnFe-LDHzyme/4-phenyl urazole catalytic system^a^

Entry	Substrate	Product	DDQ		4-Phenyl urazole	
			Time (h)	Yield (%)	Time (h)	Yield (%)
1	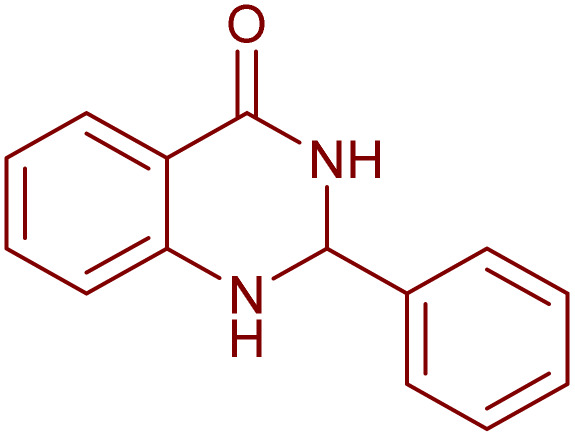	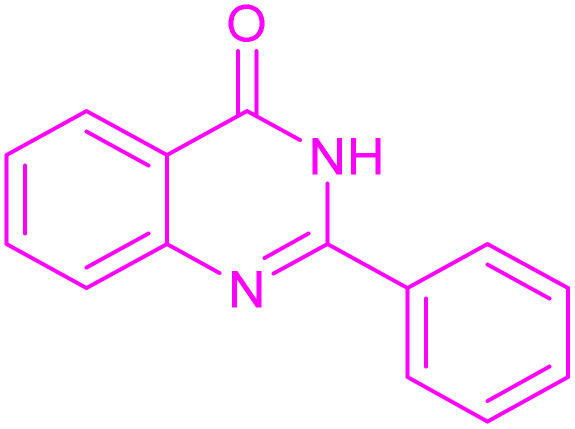	18	98	20	98
2	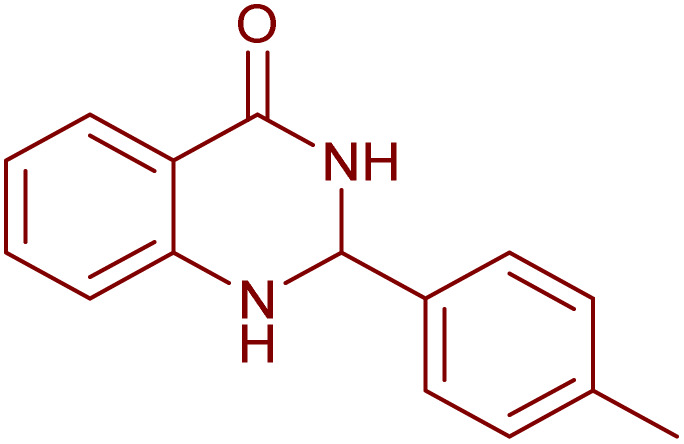	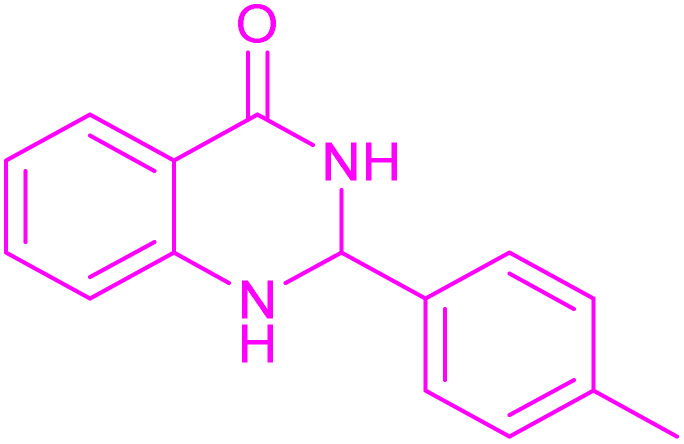	17	98	18	96
3	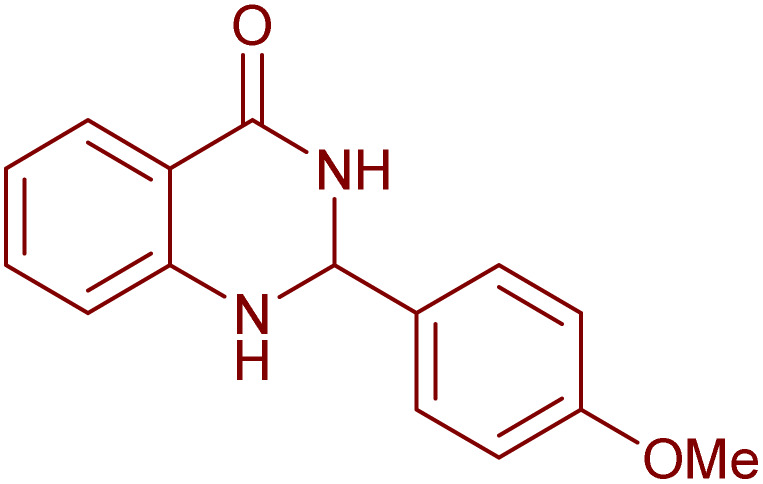	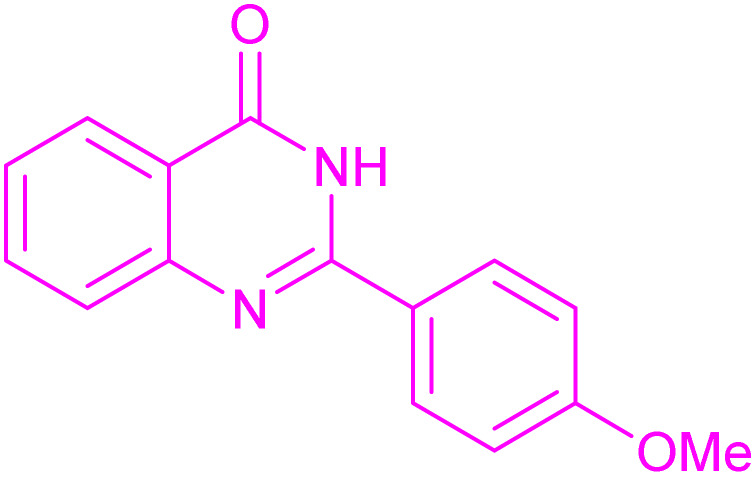	16.5	98	17	98
4	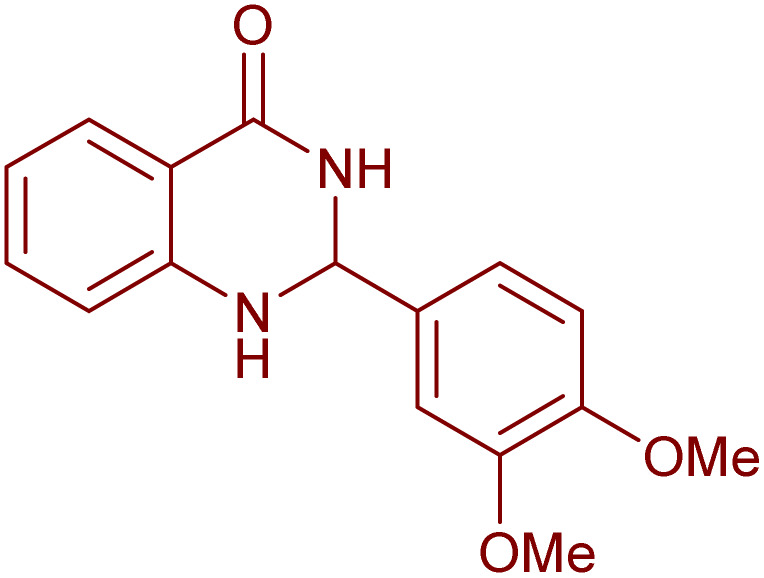	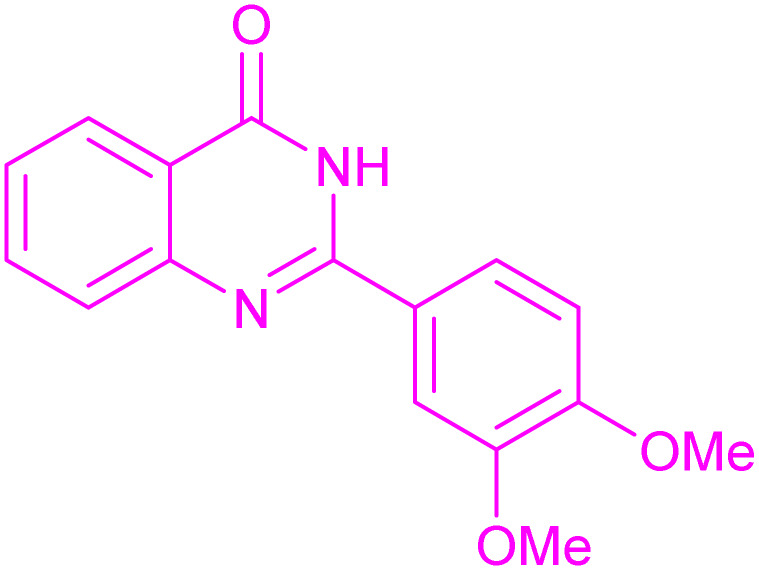	18	92	18.5	85
5	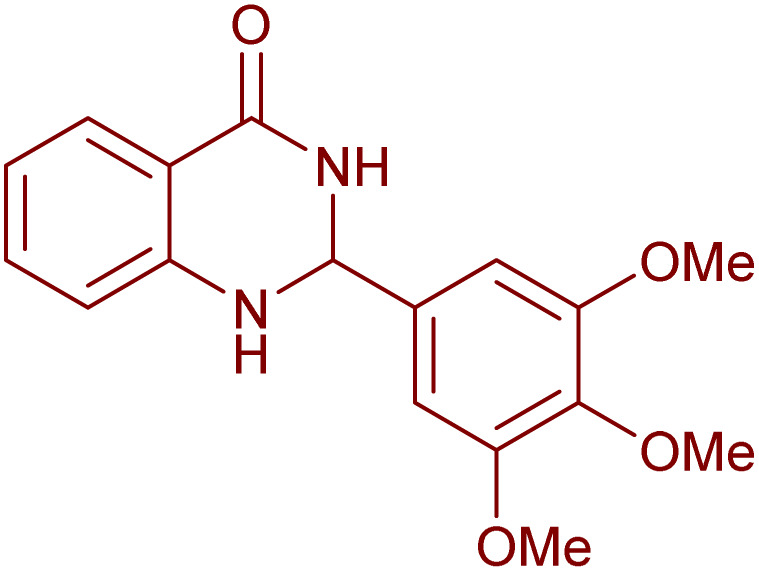	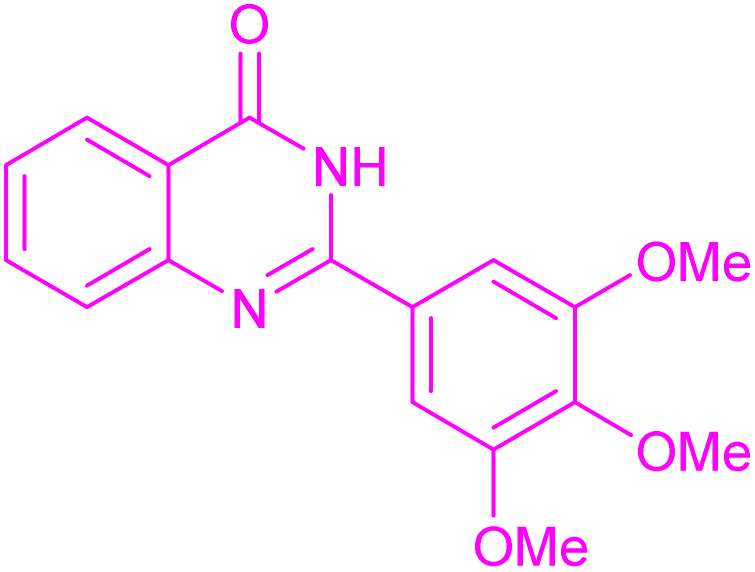	18	89	20	82
6	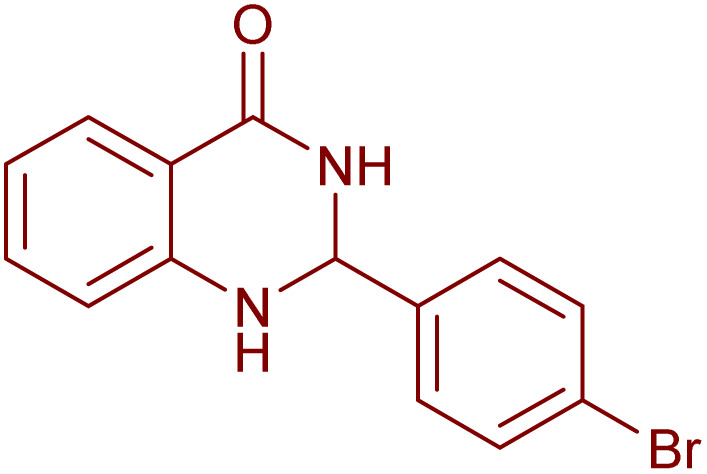	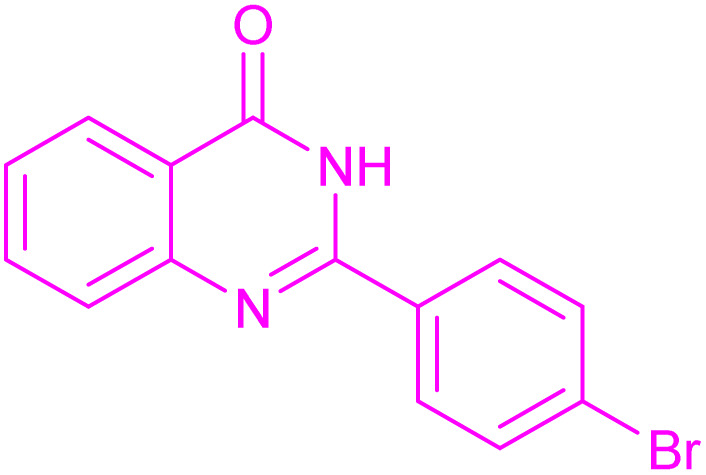	18	85	19.5	82
7	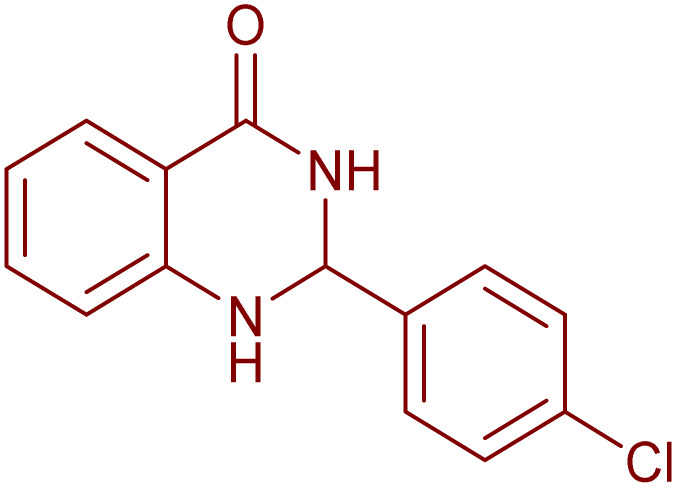	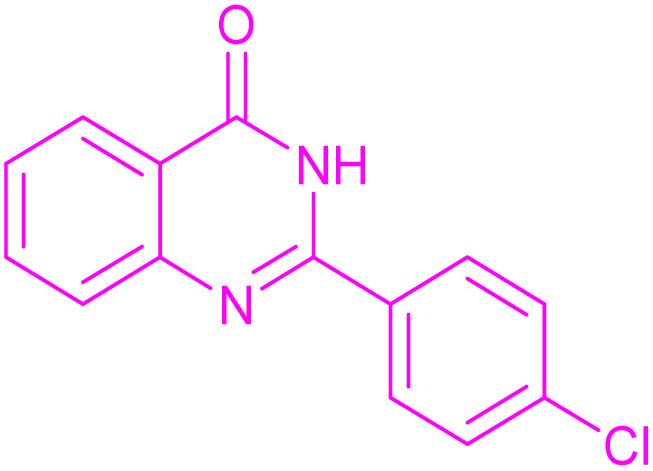	19	85	20	80
8	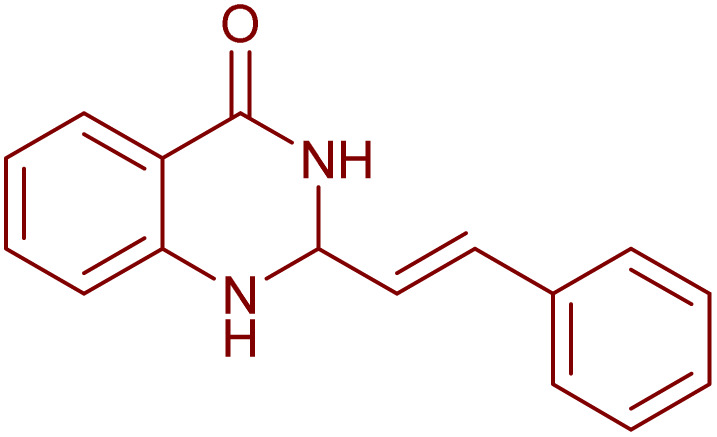	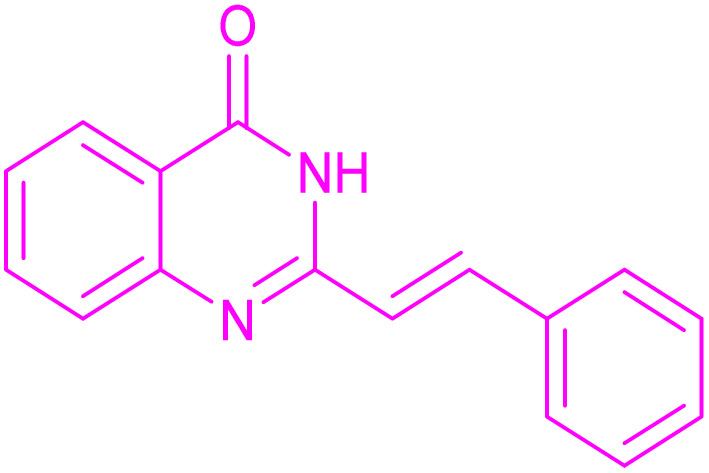	23	24	77	80
9	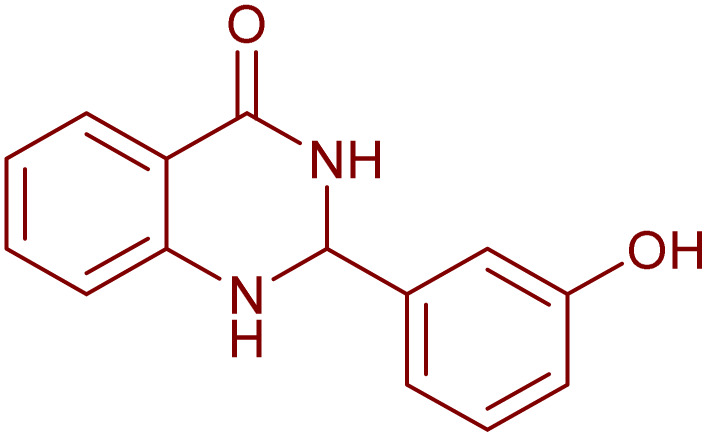	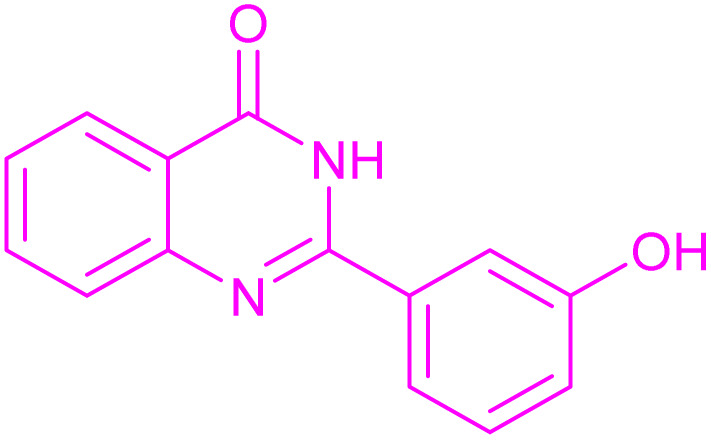	22	75	24	70
10	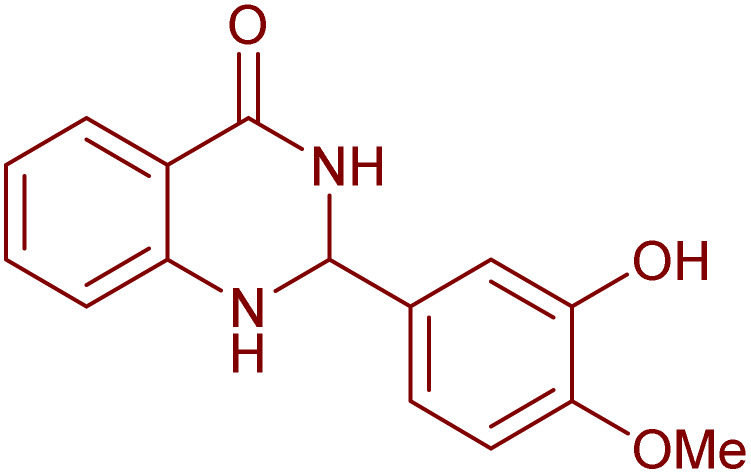	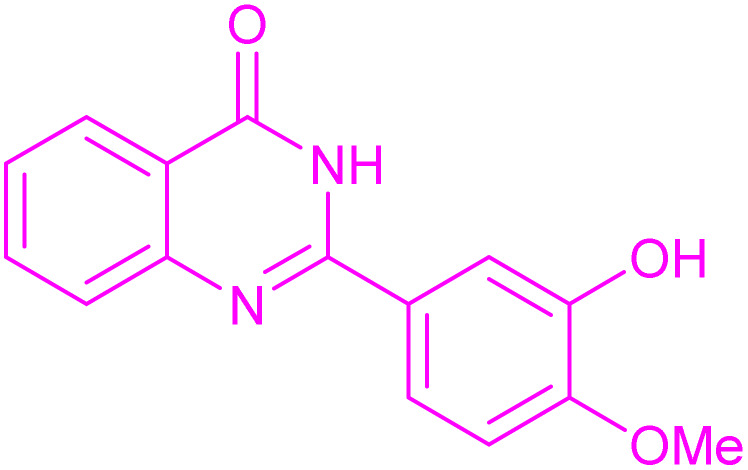	22	70	24	68

a Reaction Conditions: substrate (1 mmol), CuMnFe-LDHzyme (140 mg), mediator (10 mol%), CH_3_CN (3 mL) under O_2_ (balloon).

Having successfully achieved the aerobic oxidative synthesis of 2-substituted quinazolines, we expanded the application of CuMnFe-LDHzyme/DDQ and CuMnFe-LDHzyme/4-phenyl urazole catalytic systems for aerobic oxidation of Hantzsch 1,4-dihydropyridines to pyridines. The effect of the amount of CuMnFe-LDHzyme and DDQ or 4-phenyl urazole, solvent, and temperature on the oxidation reaction of diethyl 2,6-dimethyl-4-phenyl-1,4-dihydropyridine-3,5-dicarboxylate to diethyl2,6-dimethyl-4-phenyl-3,5-pyridinedicarboxylate as a model reaction was investigated (Table 4).

**Table 4:** Optimization of reaction conditions for aerobic dehydrogenation of 1,4-dihydropyridines with CuMnFe-LDHzyme^a^

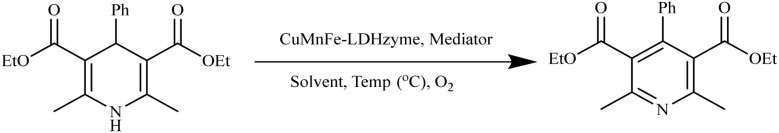					
Entry	Mediator (mol%)	Amount of CuMnFe-LDHzyme	Temperature (°C)	Solvent	Yield (%)
1	—	130	50	CH_3_CN	55
2	DDQ (5)	130	50	CH_3_CN	55
3	4-Phenyl urazole (5)	110	50	CH_3_CN	58
**4**	**DDQ (10)**	**130**	**50**	**CH**_**3**_ **CN**	**95**
**5**	**4-Phenyl urazole (10)**	**110**	**50**	**CH**_**3**_ **CN**	**95**
6	DDQ (10)	—	50	CH_3_CN	45
7	4-Phenyl urazole (10)	—	50	CH_3_CN	Trace
8	DDQ (10)	100	50	CH_3_CN	70
9	DDQ (10)	120	50	CH_3_CN	83
10	4-Phenyl urazole (10)	100	50	CH_3_CN	85
11	DDQ (10)	130	50	H_2_O	35
12	DDQ (10)	130	50	DMSO	45
13	4-Phenyl urazole (10)	110	50	H_2_O	37
14	4-Phenyl urazole (10)	110	50	DMSO	60
15	DDQ (10)	130	40	CH_3_CN	70
16	4-Phenyl urazole (10)	110	40	CH_3_CN	60

a Reaction conditions: CuMnFe-LDHzyme (130 mg), substrate (1 mmol), mediator (10 mol%), solvent (3 mL), molecular oxygen (balloon) 20 h; the bold represented the most effective reaction conditions.

Accordingly, CuMnFe-LDHzyme(130 mg)/DDQ(10 mol%) or CuMnFe-LDHzyme(110 mg)/4-phenyl urazole (10 mol%) under O_2_ in CH_3_CN (3 mL) mixture at 50 °C was found to be ideal for complete conversion of 1,4-dihydropyridines to the corresponding pyridines (Table 4, entries 4 and 5). When the amount of CuMnFe-LDHzyme or mediator (DDQ or 4-phenyl urazole) was reduced, the yield dropped (Table 4, entries 1–2 and 8–10). By decreasing the reaction temperature to 40 °C, the lower yield was observed (Table 4, entries 15 and 16).

To generalize the scope of the reaction, a series of structurally diverse Hantzsch 1,4-dihydropyridines were subjected to aerobic oxidation under the optimized reaction conditions, and the results are presented in Table 5.

**Table 5:** Aerobic oxidative synthesis of pyridine derivatives with CuMnFe-LDHzyme/DDQ^a^ and CuMnFe-LDHzyme/4-phenyl urazole^b^ catalytic system

Entry	Substrate	Product	DDQ		4-Phenyl urazole	
			Time (h)	Yield (%)	Time (h)	Yield (%)
1	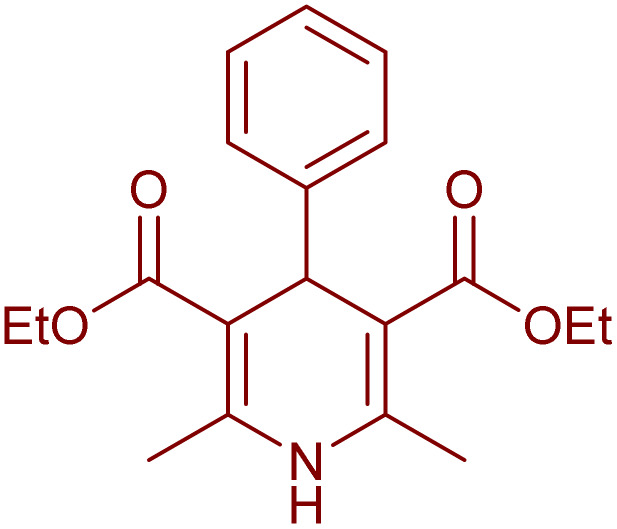	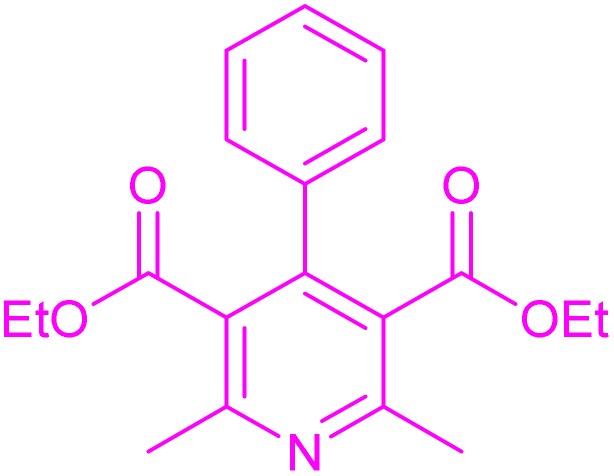	18	98	15	98
2	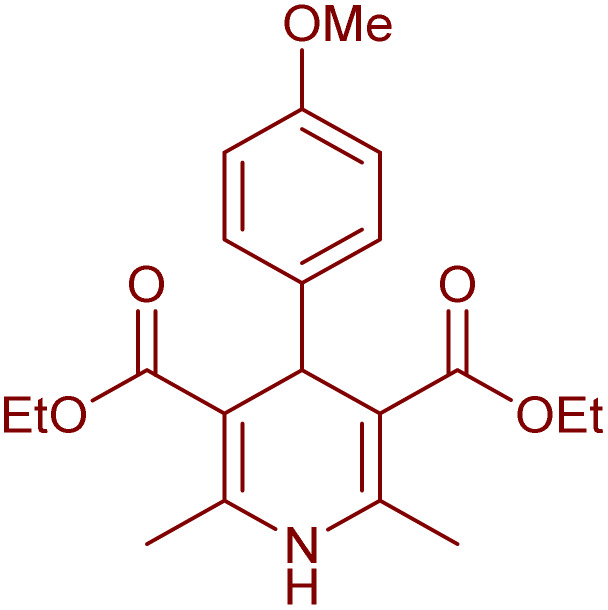	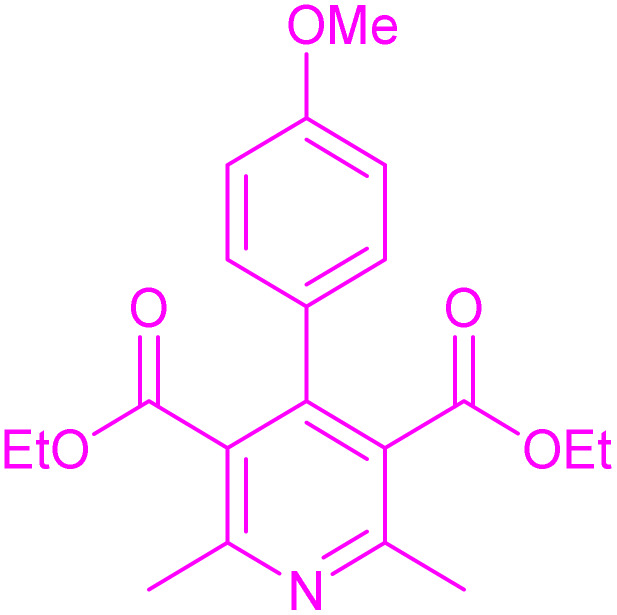	16	98	14	98
3	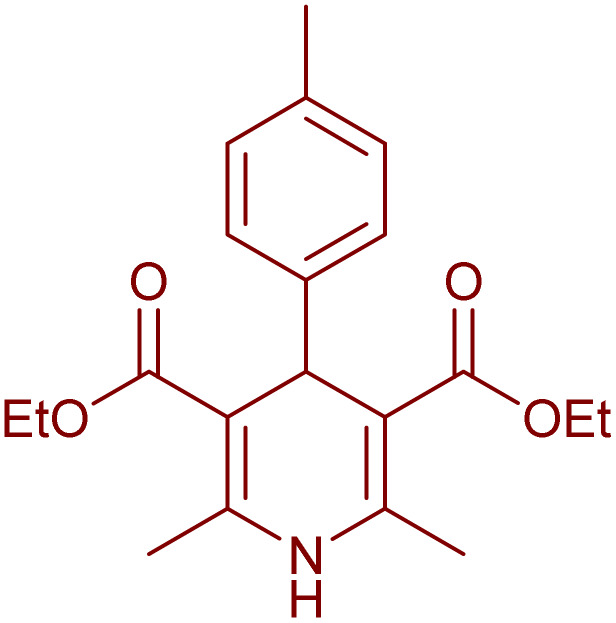	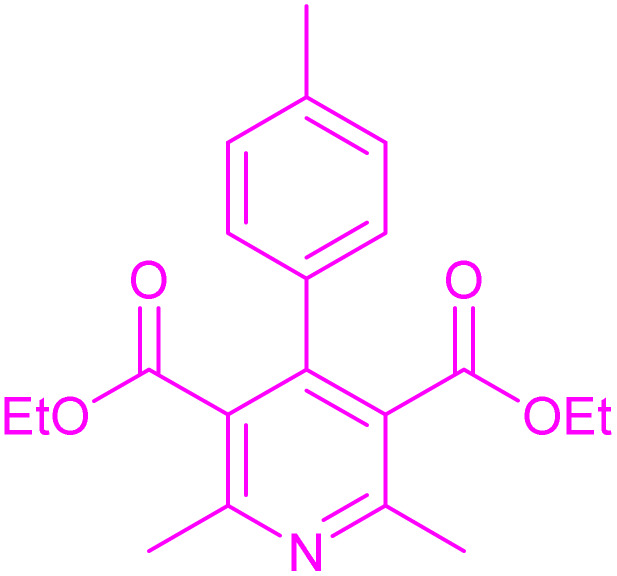	16	98	14	98
4	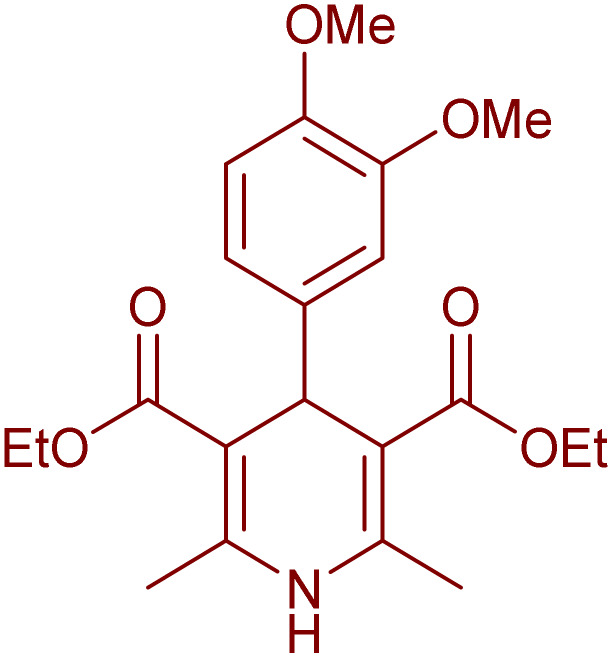	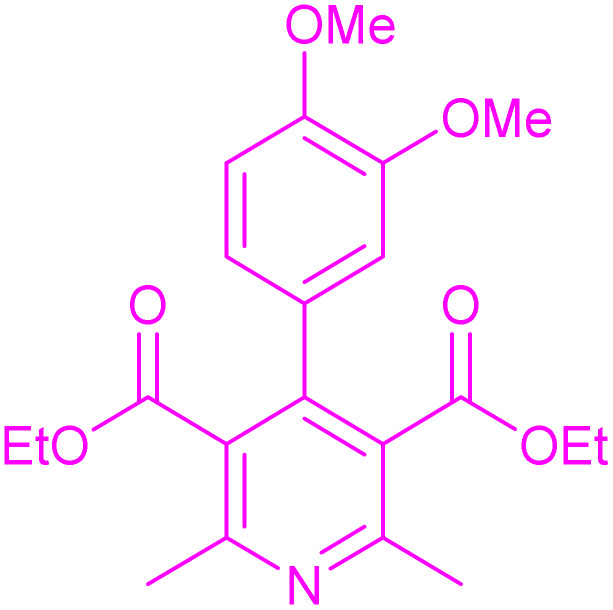	16	90	14.5	95
5	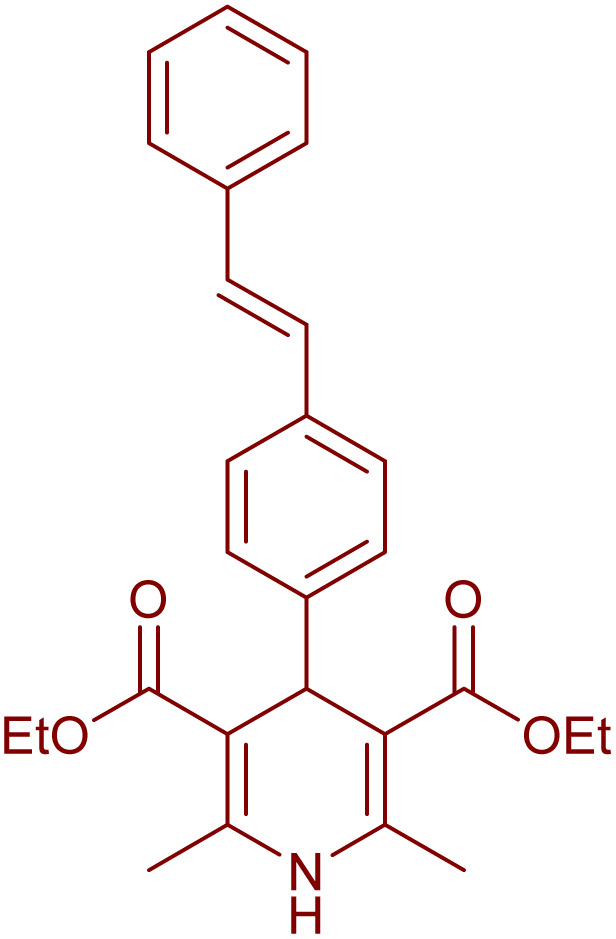	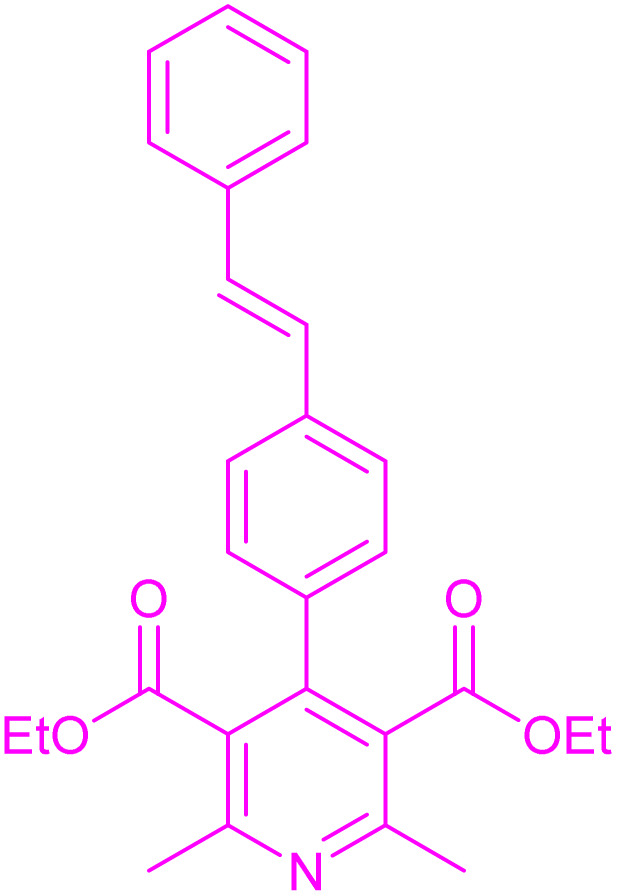	20	60	19	68
6	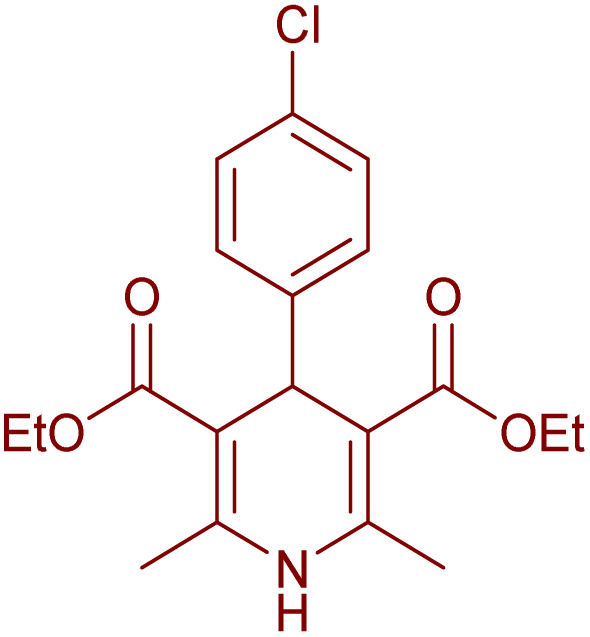	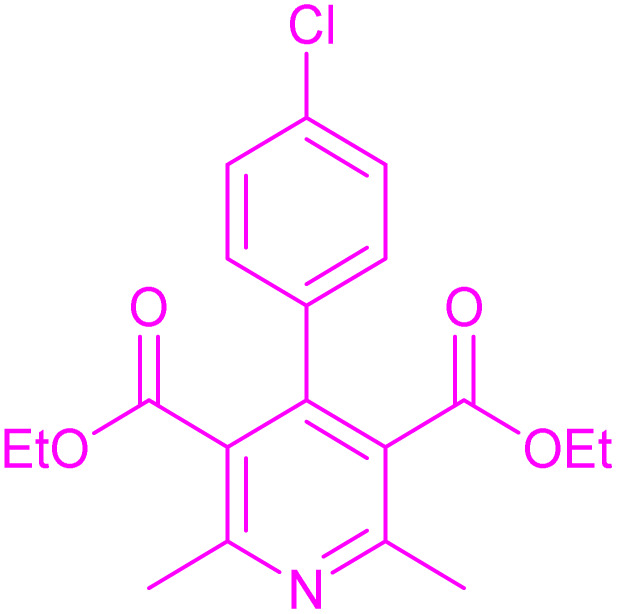	23	76	21	85
7	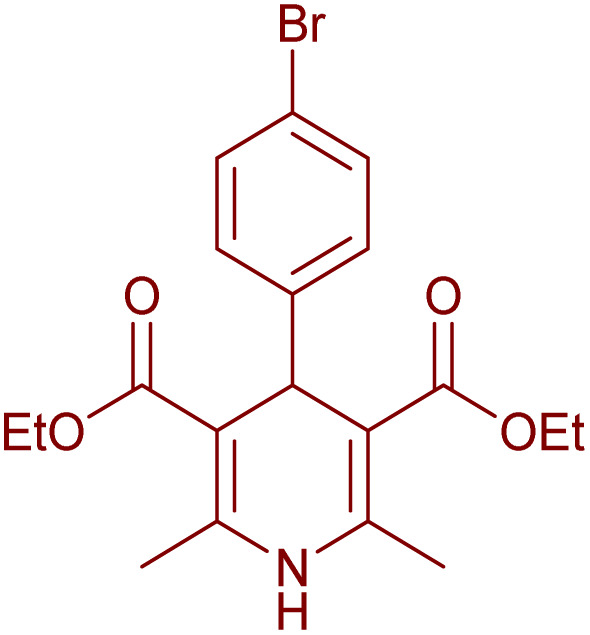	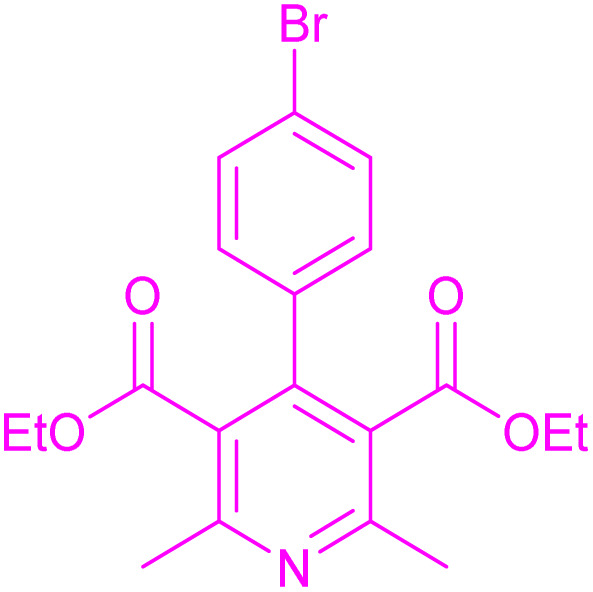	23	80	20	90
9	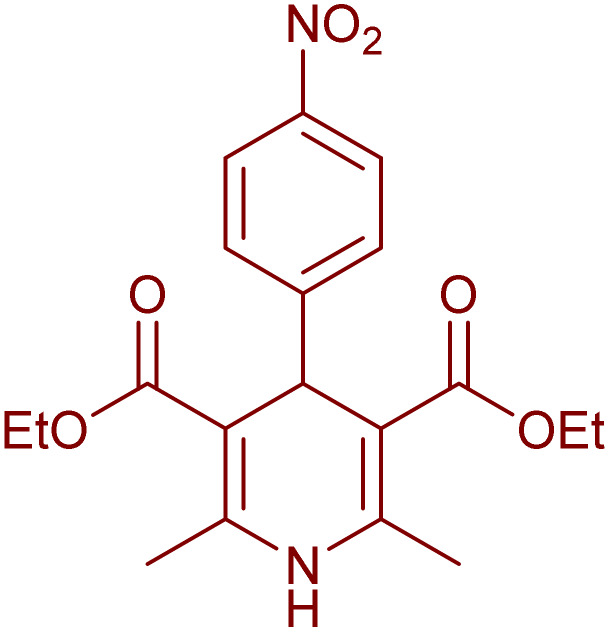	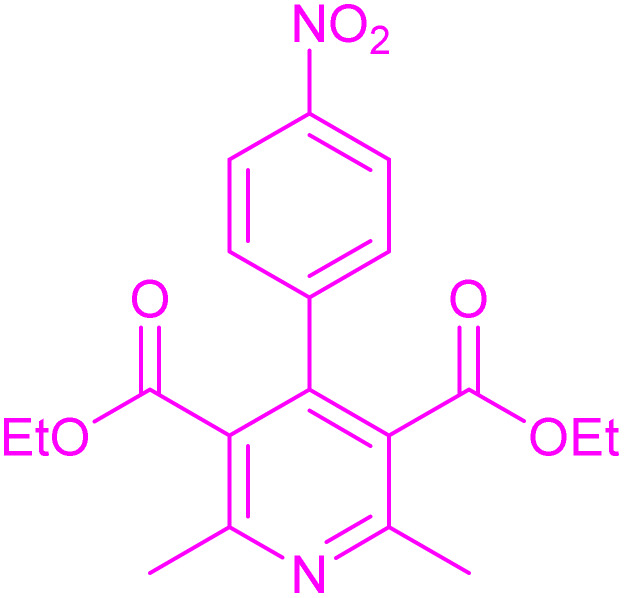	24	53	24	68

a Reaction conditions: substrate (1 mmol), CuMnFe-LDHzyme (130 mg), DDQ (10 mol%), CH_3_CN (3 mL) under O_2_ (balloon).

b Substrate (1 mmol), CuMnFe-LDHzyme (110 mg), 4-phenyl urazole (10 mol%), CH_3_CN (3 mL) under O_2_ (balloon).

**Fig. 2:**
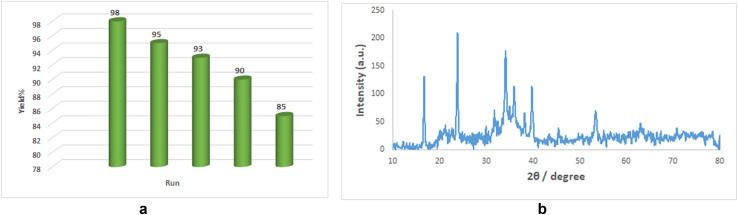
(a) Relative activity of theCuMnFe-LDHzyme evaluated in the aerobic oxidation of 2-phenyl-2,3-dihydroquinazolin-4(1*H*)-one reaction during the recycling and reuse processes. (b) XRD patterns of the CuMnFe-LDHzyme after the reaction.

The catalytic activity of CuMnFe-LDHzyme remains remarkably stable, sustaining its efficacy even after months of storage conditions. This remarkable stability stands in stark contrast to natural laccase losing over 50% of its catalytic activity when stored at room temperature for just five days. The recyclability and reusability of the CuFeMnLDHzyme were evaluated in the aerobic oxidation of 2-phenyl-2,3-dihydroquinazolin-4(1*H*)-one under optimized conditions (Table 2, entry 2). After the reaction was completed, the CuFeMnLDHzyme was recovered by centrifugation (3000 rpm for 3 minutes), thoroughly washed with acetonitrile (3 × 5 mL), and reused for multiple catalytic cycles. The CuFeMnLDHzyme retained 85% of its catalytic activity after five cycles (Fig. 2a). Furthermore, there was no significant change in the structure of the LDHzyme (Fig. 2b). These results indicate that the CuFeMnLDHzyme demonstrates greater catalytic stability and recyclability compared to natural laccase.

**Scheme 1:**
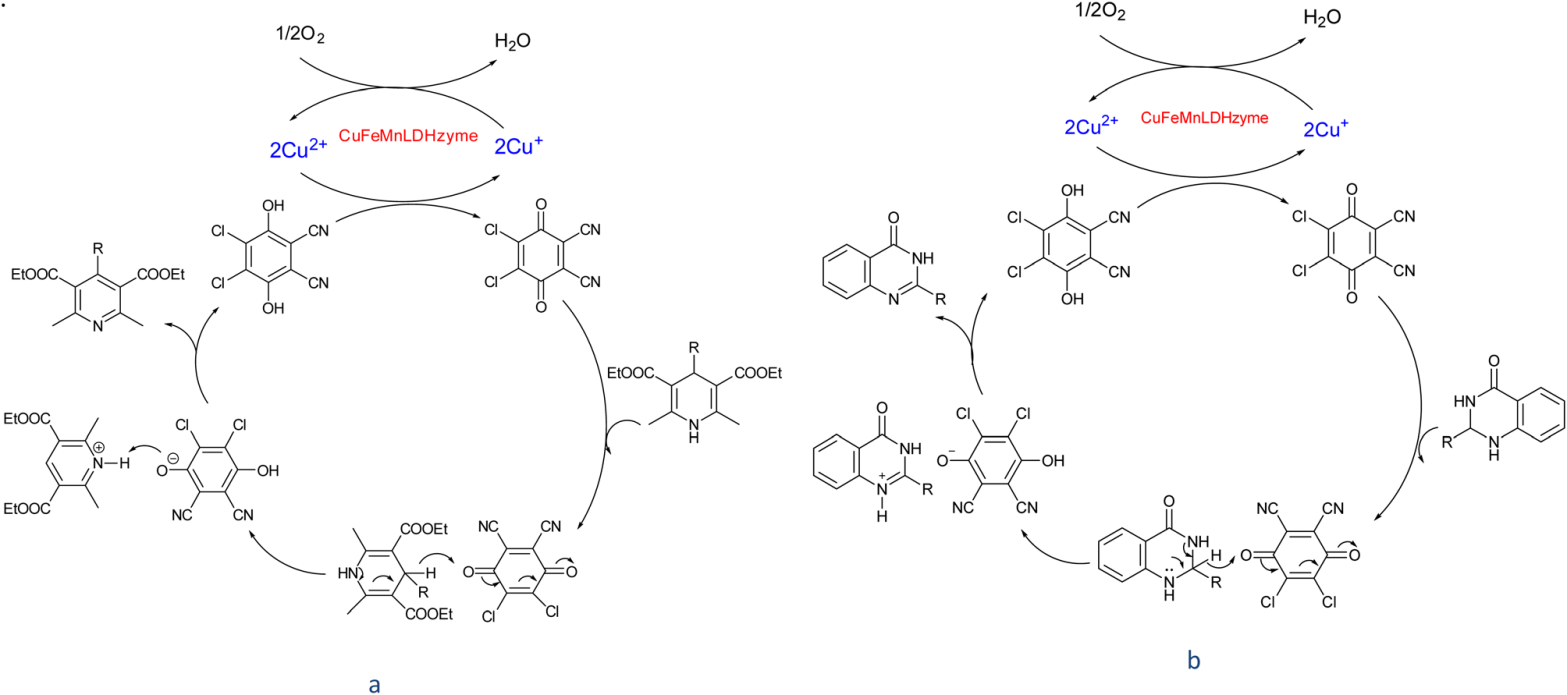
Proposed mechanism for the synthesis of pyridines (a) and quinazolinones (b) in the presence of the CuMnFe-LDHzyme/DDQ.

Although the precise mechanisms governing the reactions remain ambiguous at this time, previous studies concerning the utilization of DDQ and 4-phenyl urazole in the aerobic oxidation of organic compounds facilitated by laccase^47,48^ provide valuable insights. The oxidation of the substrate is initiated by hydride transfer to DDQ *via* an anomeric oxidation pathway,^49^ leading to the formation of a substrate-cation/DDQH^−^ ion pair complex. This intermediate is then converted to the desired product along with DDQH_2_. Subsequently, the by-product DDQH_2_ is re-oxidized by a CuMnFeLDHzyme catalyst. Finally, the reduced LDH catalyst is re-oxidized by molecular oxygen, completing the catalytic cycle shown in Scheme 1.

**Scheme 2:**
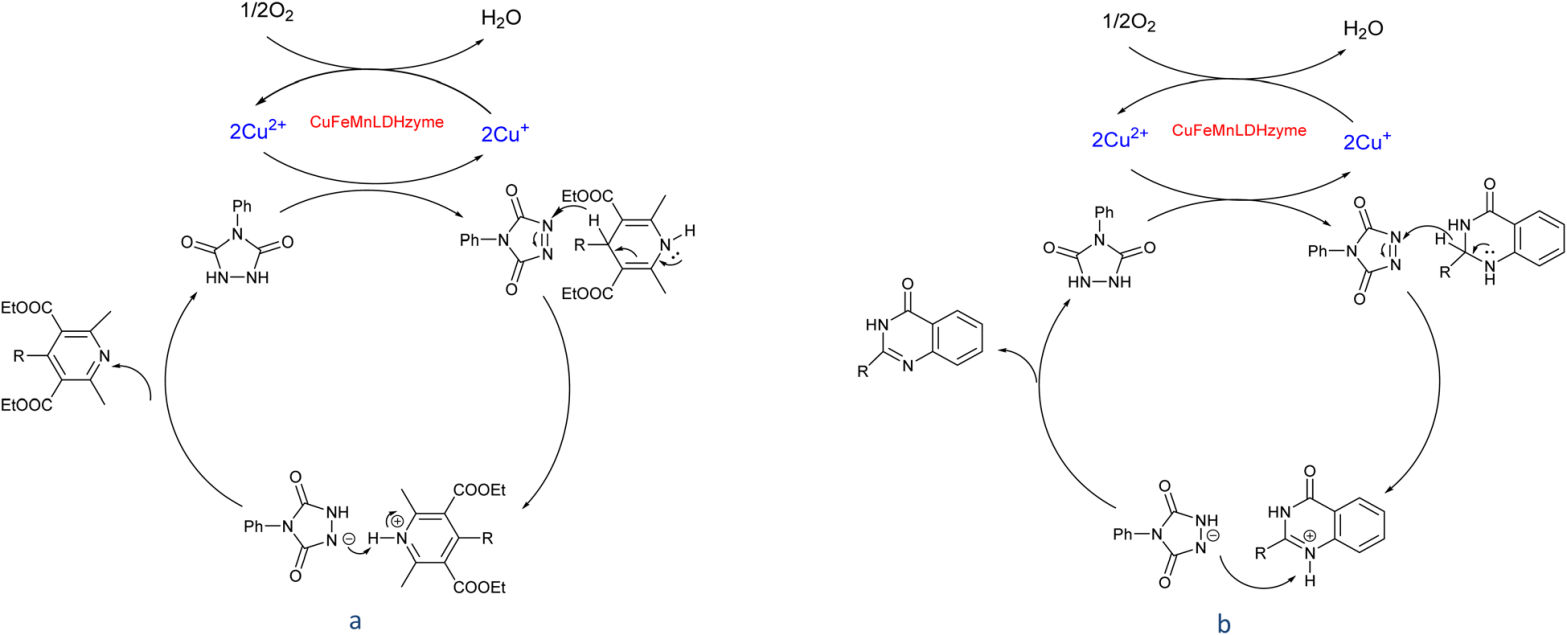
Proposed mechanism for the synthesis of pyridines (a) and quinazolinones (b) in the presence of the CuMnFe-LDHzyme/4-phenyl urazole.

Scheme 2 shows the proposed mechanism for the aerobic oxidation of 2,3-dihydroquinazolin-4(1*H*)-ones and 1,4-dihydropyridines in the presence of CuFeMnLDHzyme/4-phenyl urazole catalytic system, initially, CuFeMnLDHzyme oxidize 4-phenyl urazole to 4-phenyl-1,2,4-triazole-3,5-dione (TAD) by losing H_2_ by. The oxidized mediator (TAD) then acts as an oxidizing agent by abstracting hydride from the substrates, which contributes to the formation of a cation intermediate. Subsequently, the deprotonation this intermediated by 4-phenyl urazole anion completes the oxidation reaction and regenerate 4-phenyl urazole.^50^ These cycles are inspired by the natural laccase mechanism, where the role of the active Cu^2+^ in laccase is mimicked by the Cu sites in the LDH structure, while the presence of Fe and Mn enhances the stability and overall efficiency of the process.

To demonstrate the efficiency of the CuMnFe-LDHzyme/DDQ and CuMnFe-LDHzyme/4-phenylurazole catalyst systems as Laccase Mimicking Nanozymes, we compared our results on the dehydrogenation of 2-phenyl-2,3-dihydroquinazolin-4(1*H*)-one and diethyl 2,6-dimethyl-4-phenyl-1,4-dihydropyridine-3,5-dicarboxylate with findings from some previous studies utilizing laccase catalytic systems. The details are presented in Table 6.

**Table 6:** Comparison of the CuMnFe-LDHzyme catalyst system with the laccase catalyst system for the aerobic oxidation

Entry	Catalyst	Reaction conditions	Time(h)	Isolated yield (%)	Reference
1	Laccase (174 mg, 200 U)/DDQ (20 mol%)	NaPBS/CH_3_CN, O_2_ or air, 45°C	24	90	51
2	Laccase (174 mg, 200 U)/4-phenyl urazole	NaPBS/CH_3_CN, O_2_, 40 °C	22	99	43
3	CuMnFe-LDHzyme(140 mg)/DDQ or 4-phenyl urazole (10 mol%)	CH_3_CN, O_2_ or air, 60 °C	18	98	This work
4	CuMnFe-LDHzyme(110 mg)/4-phenyl urazole (10 mol%)	CH_3_CN, O_2_, 50 °C	15	98	This work
5	CuMnFe-LDHzyme(140 mg)/DDQ (10 mol%)	CH_3_CN, O_2_, 50 °C	18	98	This work

## 3. Experimental

### 3.1. Material and physical measurements

All chemical substances, reagents, and solvents, with the exception of *N*-heterocyclic compounds, were procured from the Merck and Aldrich Chemical Companies and utilized without undergoing any additional purification processes. FT-IR and ^1^H, spectra were acquired utilizing the Thermo Nicolet Nexus 670 and Bruker Avance spectrometers (500 MHz). Melting points were measure on a Barnstead Electrothermal IA 9100 and are uncorrected. The dimensions of the particles were ascertained *via* SEM using FESEM. The chemical composition of the prepared nano catalyst was measured by EDX (Energy Dispersive X-ray Spectroscopy) and ICP-MSI.

### 3.2. Preparation of LDHzymes

LDHzymes were firstly synthesized according to a previously reported method.^44^In a typical experiment, the mixed metal salt solution was first prepared (Table 7) subsequently followed, sodium hydroxide (0.12 mol) and sodium carbonate (0.01 mol) were combined in 60 mL of deionized water to generate an alkaline solution. The metal salt solution, totaling 60 mL, was meticulously introduced into the prepared alkaline solution through dropwise addition within a 1000 mL flask. The pH of the resulting amalgamation was required to be adjusted to a value of 10 through the careful application of either sodium hydroxide or hydrochloric acid solution (0.1 M), while the reaction temperature was to be maintained at 60 °C throughout the synthesis period of 24 hours. Subsequently, the resultant product underwent filtration and was washed with an appropriate volume of deionized water utilizing suction filtration to eliminate any impurities. Ultimately, the solid residue was subjected to drying at 70 °C and subsequently ground into a fine powder.

**Table 7:** The amount of each metal ion in the LDHzymes preparation

LDHzymes	Cu (NO_3_)_2_·3H_2_O	Mn (NO_3_)_2_·4H_2_O	Fe (NO_3_)_3_·9H_2_O	Zn (NO_3_)_2_·6H_2_O
CuZnFe-LDH	0.5 mol l^−1^	—	0.25 mol l^−1^	0.25 mol l^−1^
CuMnFe-LDH	0.5 mol l^−1^	0.25 mol l^−1^	0.25 mol l^−1^	—
CuFe-LDH	0.25 mol l^−1^	—	0.25 mol l^−1^	—
MnFe-LDH	—	0.25 mol l^−1^	0.25 mol l^−1^	—
CuMn-LDH	0.25 mol l^−1^	0.25 mol l^−1^	—	—

### 3.3. General procedure for the aerobic oxidation of 2,3-dihydroquinazolin-4(1*H*)-one derivatives

2,3-dihydroquinazolin-4(1*H*)-one was synthesized following the methodology previously documented^52^ through the reaction of o-anthranilamide with aldehyde in the presence of sulfamic acid for 30 min at room temperature^52^ Subsequent to the primary cyclization, the step involves the aerobic oxidation of the synthesized 2,3-dihydroquinazolin-4(1*H*)-one. This transformation is promoted by the addition of a 2,3-dihydroquinazolin-4(1H)-one (1 mmol), CuMnFe-LDHzyme (140 mg), and DDQ or 4-phenyl urazole (10 mol%) to a solution of acetonitrile (2 mL) and the reaction mixture was stirred under O_2_ (balloon) at a temperature of 60 °C for the duration specified in Table 3. The CuFeMnLDHzyme was removed by filtration. The mixture was then treated with a 10% (w/v) aqueous NaOH solution and extracted with ethyl acetate (3 × 10 mL). The combined organic phases were dried over anhydrous Na_2_SO_4_, filtered, and concentrated under reduced pressure to yield the pure product. The crude product was subsequently purified using column chromatography on silica gel, employing a solvent mixture of *n*-hexane and ethyl acetate in a 75 : 25 volumetric ratio. The products were identified by comparing their NMR spectra and melting points with those of authentic samples.^34,53^

### 3.4. General procedure for the aerobic oxidation of Hantzsch ester 1,4-DHPs

1,4-Dihydropyridine derivatives were synthesized following the previously documented methodology^54^ through the reaction of ethyl acetoacetate, a suitable aldehyde, and ammonium acetate. Next, to a solution of acetonitrile (2 mL) containing 1 mmol of the synthesized 1,4-dihydropyridine compound, either CuMnFe-LDHzyme (130 mg) and DDQ (10 mol%) or CuMnFe-LDHzyme (110 mg) and 4-phenyl urazole (10 mol%) were added. The resulting mixture was stirred at 50 °C in the presence of molecular oxygen (provided *via* a balloon) for the duration specified in Table 5. Upon completion of the reaction, as monitored by TLC, the CuMnFe-LDHzyme was removed by filtration. The mixture was then treated with a 10% (w/v) aqueous NaOH solution and extracted with ethyl acetate (3 × 10 mL). The combined organic phases were dried over anhydrous Na_2_SO_4_, filtered, and concentrated under reduced pressure to yield the pure product. The crude product was subsequently purified using column chromatography on silica gel, employing a solvent mixture of *n*-hexane and ethyl acetate in a 75 : 25 volumetric ratio. The products were identified by comparing their NMR spectra and melting points with those of authentic samples.^55,56^

## 4. Conclusion

In summary, CuMnFe-LDHzyme was utilized as Laccase Mimicking Nanozymes in the aerobic oxidation of 2,3-dihydroquinazolin-4(1*H*)-ones and 1,4-dihydropyridines. The notable benefits of these methodologies are delineated as follows: (i) the CuMnFe-LDHzyme can function as a laccase enzyme compared to the natural counterpart, the fabricated enzyme mimic possesses high stability, high activity, easy production procedures, low cost, reusability, and versatility. Furthermore, the capacity of CuMnFe-LDHzyme to preserve its catalytic functionalities under extreme conditions characterized by elevated pH values, high thermal environments, or the presence of denaturing substances represents a substantial progression in the field of enzyme technology; (ii) the use of air or O_2_ as an environmentally benign, inexpensive and abundant oxidant and the formation of H_2_O as the only nontoxic by-product; (iii) the synthesis of structurally diverse quinazolinones and pyridines in good to high yields.

## Conflicts of interest

There are no conflicts to declare.

## Supplementary Material

RA-015-D5RA03505H-s001

## Data Availability

The data supporting this article have been included as part of the supplementary information (SI). Supplementary information is available: the ^1^H NMR spectra of selected products are included. See DOI: https://doi.org/10.1039/d5ra03505h.
