# Supplementary material for: Application of layered double hydroxides as laccase mimicking nanozymes in the oxidation of 2,3-dihydroquinazolin-4(1H)-ones and hantzsch 1,4-dihydropyridines
Source: RSC Adv. 2025 Sep 30;15(43):36237–48. doi: 10.1039/d5ra03505h (PMC12481237; doi:10.1039/d5ra03505h)
Supplement: RA-015-D5RA03505H-s001 [file RA-015-D5RA03505H-s001.pdf]

Supplementary Information (SI) for RSC Advances.
This journal is © The Royal Society of Chemistry 2025

Supporting Information

Application of layered double hydroxides as laccase mimicking nanozymes in the aerobic oxidation of 2,3-dihydroquinazolin-4(1H)-ones and hantzsch 1,4-dihydropyridines

Nadia Ghorashi[a] and Amin Rostami *[a]

[a] *Department of Chemistry, Faculty of Science, University of Kurdistan, 66177-15175, Sanandaj, Iran.*
E-mail: a.rostami@Uok.ac.ir; Fax: +988716624004; Tel: +989183730910

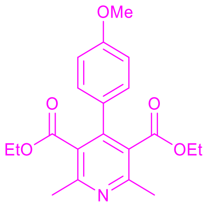

Diethyl 1,6-Dimethyl-4-(*p*-methoxyphenyl) pyridine-3,5-dicarboxylate

^{1}H NMR (500 MHz, $CDCl_3$): δ 7.19 (d, *J* = 8.0 Hz, 2 H), 6.86 (d, *J* = 8.0 Hz, 2 H), 4.03 (q, *J* = 7.1 Hz, 4H), 3.79 (s, 3H), 2.58 (s, 6H), 0.96 (t, *J* = 7.1 Hz, 6H).

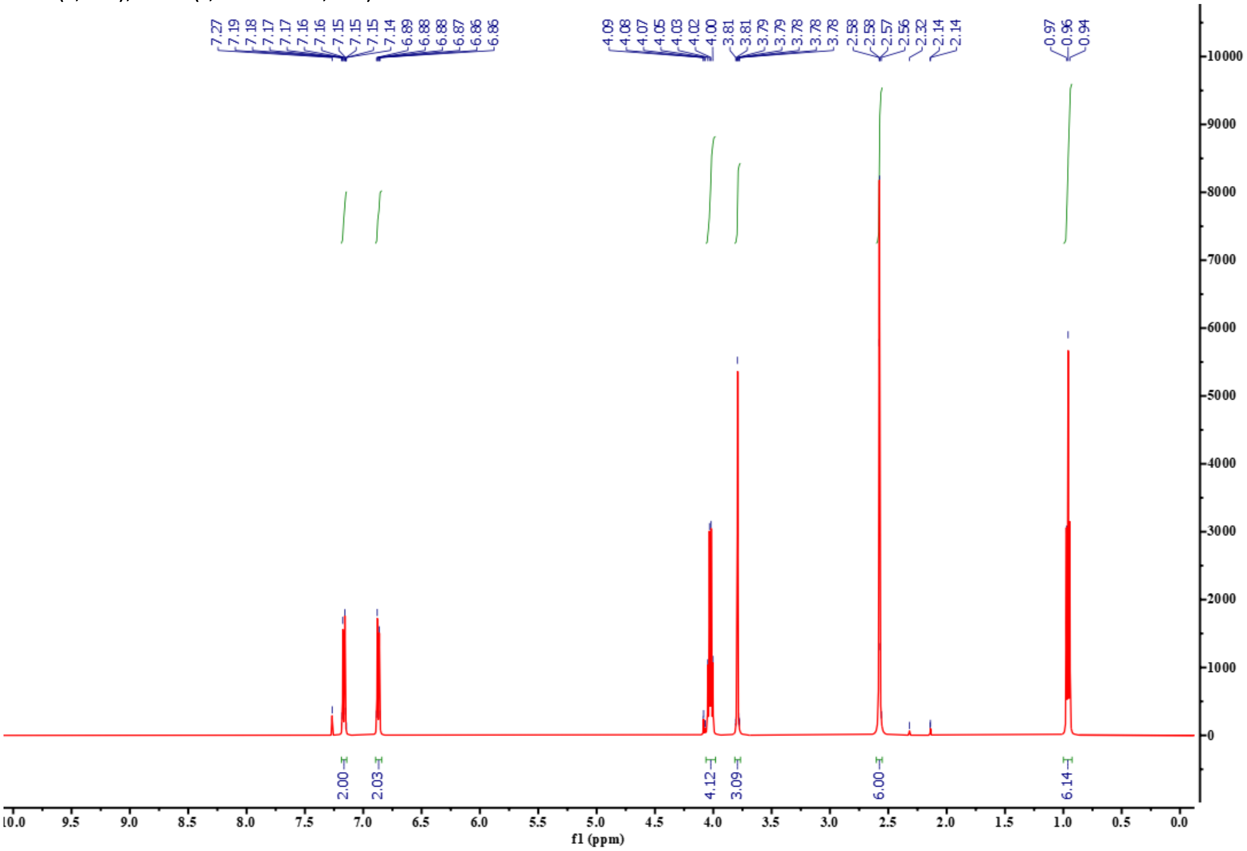

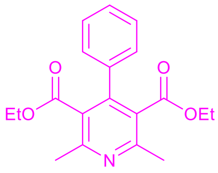

Diethyl 2,6-dimethyl-4-phenyl-3,5-pyridinedicarboxylate

^{1}H NMR (500 MHz, $CDCl_3$): δ 7.34 (t, 3H), 7.23 (d, 2H), 4.00 (q, *J* = 7.1 Hz, 4H), 2.60 (s, 6H), 0.89 (t, *J* = 7.1 Hz, 6H).

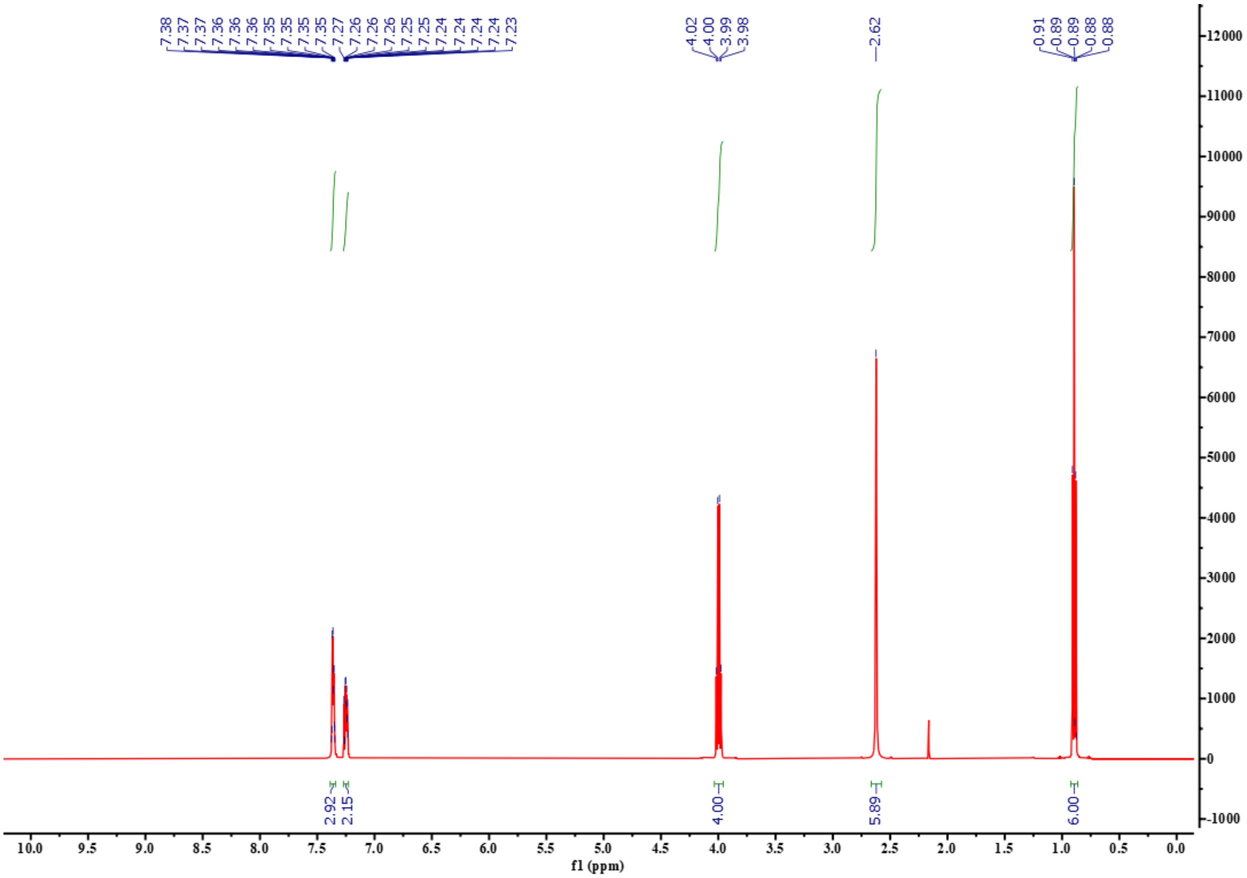

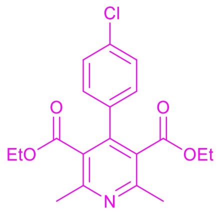

Diethyl 2,6-dimethyl-4-(4-chlorophenyl)-3,5-pyridinedicarboxylate

^{1}H NMR (500 MHz, CDCl3): δ 7.38 (d, 2H), 7.17 (d, 2H, *J* =8.4 Hz), 4.00 (q, 4H, *J* =7.2 Hz), 2.53 (s, 6H), 0.93 (t, 6H, *J* =7.2 Hz).

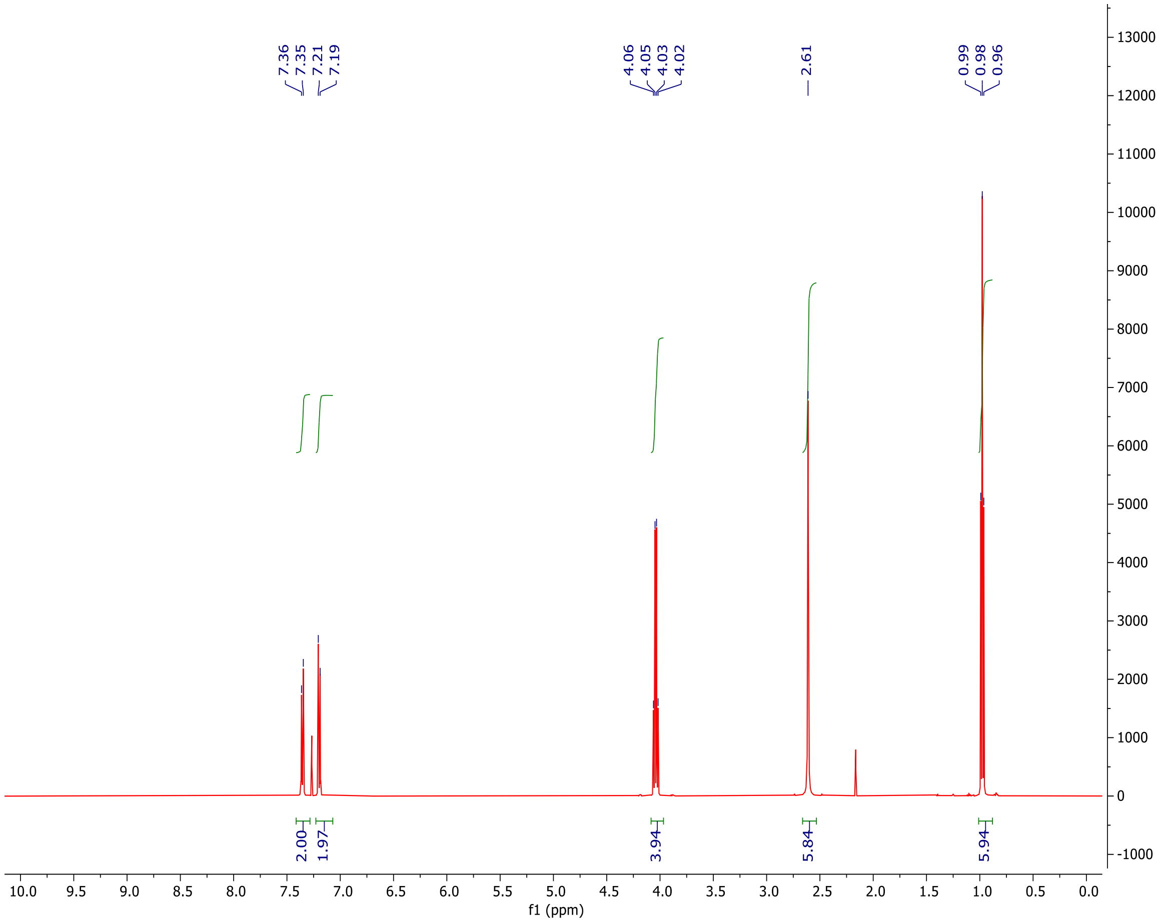

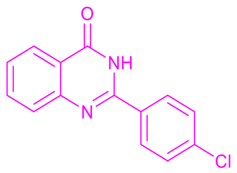

2-(4- chlorophenyl) quinazolin-4 (3H) -one

^{1}H NMR (500 MHz, DMSO): δ 12.58 (s, 1H), 8.22 (d, 2H), 8.15 (d, *J* = 7.75 Hz, 1H), 7.83 (t, *J* = 7.5 Hz, 1H), 7.76 – 7.70 (d,*J*=8.11 Hz, 1H), 7.64 (d, 2H), 7.55 – 7.50 (t, *J* = 7.34 Hz, 1H).

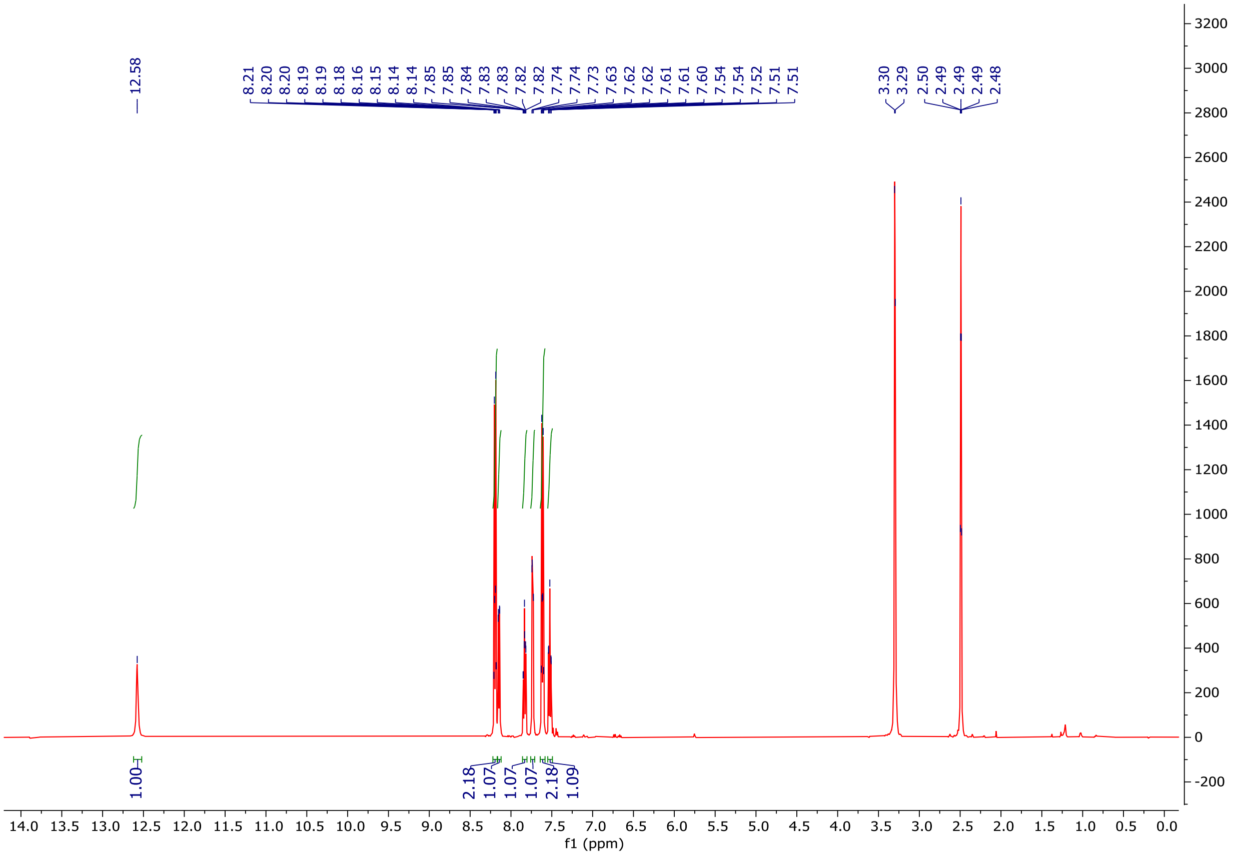

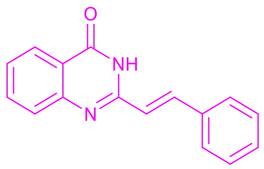

(E)-2-Styrylquinazolin-4(3H)-one

^{1}H NMR (500 MHz, DMSO): δ 12.41 (s, 1H), 8.15(d, *J* =7.20 Hz,1H) ,7.88 (d, *J* = 16.21 Hz, 1H), 7.75 (m,1H), 7.70 (m, 3H), 7.46 (m, 4H), 6.99 (d, *J* = 16.2 Hz, 1H).

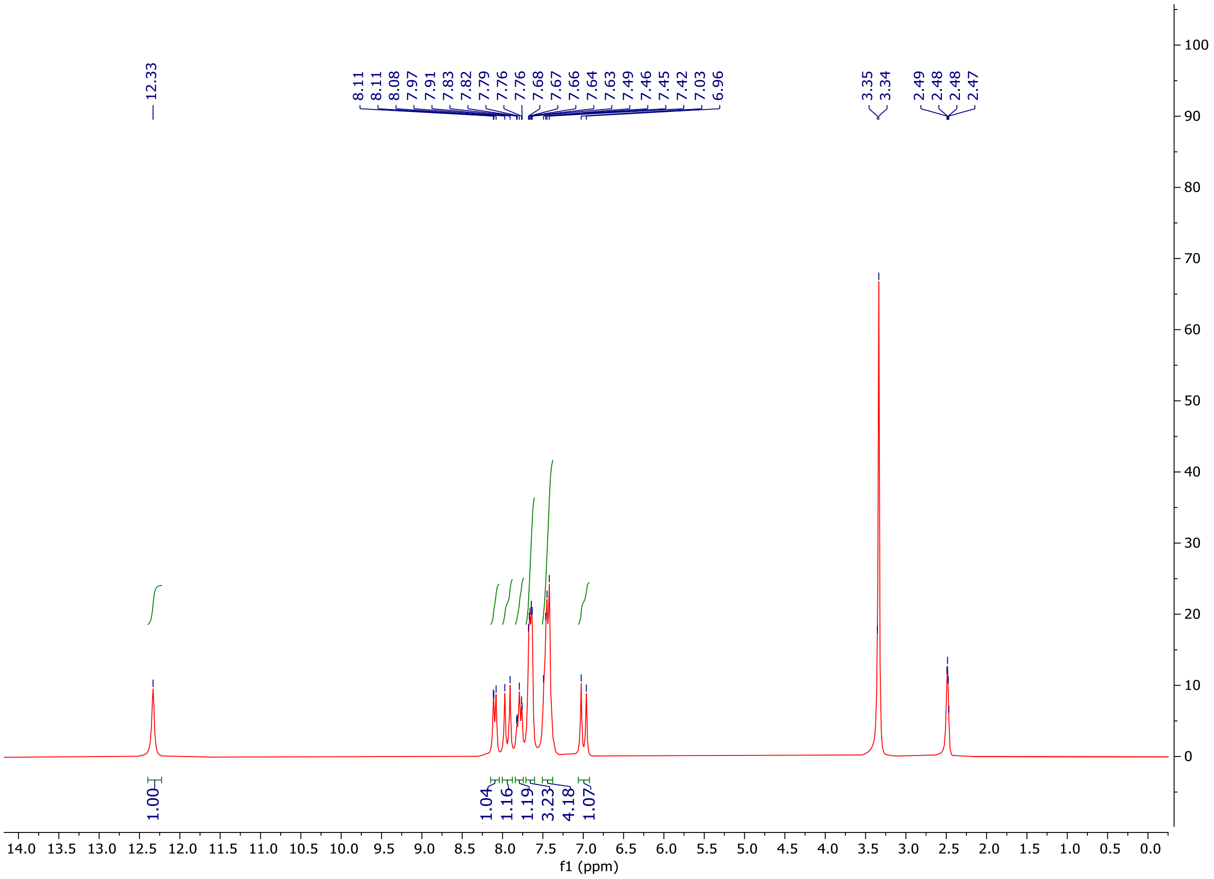

References

1. K. Upadhyaya, R. K. Thakur, S. K. Shukla and R. P. Tripathi, *The Journal of Organic Chemistry*, 2016, **81**, 5046-5055.
2. A. Sharma, J. Singh and A. Sharma, *The Journal of Organic Chemistry*, 2024, **89**, 5229-5238.
3. J. Yadav, B. Reddy, A. Basak, G. Baishya and A. V. Narsaiah, *Synthesis*, 2006, **2006**, 451-454.
